# A Novel Pear Scab (*Venturia nashicola*) Resistance Gene, *Rvn3*, from Interspecific Hybrid Pear (*Pyrus pyrifolia* × *P. communis*)

**DOI:** 10.3390/plants10122632

**Published:** 2021-11-30

**Authors:** Sewon Oh, Hyeondae Han, Daeil Kim

**Affiliations:** Department of Horticulture, Chungbuk National University, Cheongju 28644, Korea; oseng1234@cbnu.ac.kr (S.O.); hhd0916@cbnu.ac.kr (H.H.)

**Keywords:** disease resistance, genetic linkage map, InDel, ortholog, single dominant gene

## Abstract

Asian pear scab is a fungal disease caused by *Venturia nashicola*. The identification of genes conferring scab resistance could facilitate the breeding of disease-resistant cultivars. Therefore, the present study aimed to identify a scab-resistance gene using an interspecific hybrid population ((*Pyrus pyrifolia* × *P. communis*) × *P. pyrifolia*). Artificial inoculation of *V. nashicola* was carried out for two years. The segregation ratio (1:1) of resistant to susceptible individuals indicated that resistance to *V. nashicola* was inherited from *P. communis* and controlled by a single dominant gene. Based on two years phenotypic data with the Kruskal–Wallis test and interval mapping, 12 common markers were significantly associated with scab resistance. A novel scab resistance gene, *Rvn3*, was mapped in linkage group 6 of the interspecific hybrid pear, and co-linearity between *Rvn3* and one of the apple scab resistance genes, *Rvi14*, was confirmed. Notably, an insertion in pseudo-chromosome 6 of the interspecific hybrid cultivar showed homology with apple scab resistance genes. Hence, the newly discovered *Rvn3* was considered an ortholog of the apple scab resistance gene. Since the mapping population used in the present study is a pseudo-BC_1_ population, pyramiding of multiple resistance genes to pseudo-BC_1_ could facilitate the breeding of pear cultivars with durable resistance.

## 1. Introduction

*Venturia* species are a threat to stable pear (*Pyrus* spp.) production; they infect the pear leaves, branches, and fruits leading to defoliation and poor fruit quality. While fungicides restrict *Venturia* species infection, they increase management costs in pear orchards and are ineffective against the fungicide-resistant strains. Therefore, breeding a scab-resistant pear cultivar is a fundamental solution to *Venturia* species infection.

*V. nashicola* and *V. pirina* are causes of pear scab [1,2]; *V. nashicola* only infects Asian pears (*P. pyrifolia*, *P. ussuriensis*, and *P. bretschneideri*), while *V. pirina* is only pathogenic to European pears (*P. communis*). It is also well known that the Japanese indigenous pear cultivar ‘Kinchaku’ has been shown to be resistant to *V. nashicola*.

According to pathogenic characteristics, Abe et al. [3] revealed that resistance to *V. nashicola* is controlled by a single dominant gene. However, other studies have shown that multiple resistance (*R*) genes may be associated with pear scab resistance, and the genetic location of the *R* gene may differ depending on the resistant host. Terakami et al. [4] identified an *R* gene (*Vnk*) for *V. nashicola*, which is located in linkage group (LG) 1 of the indigenous pear (*P. pyrifolia* cv. Kinchaku). Besides, Cho et al. [5] reported *Rvn2*, another *R* gene to *V. nashicola*, in LG13 of an interspecific hybrid pear [(*P. pyrifolia* × *P. ussuriensis*) × *P. communis*]. They also developed cleaved amplified polymorphic sequence markers linked to the *Rvn2*. In European pears attacked by *V. pirina*, Pierantoni et al. [6] identified two major quantitative trait loci (QTLs) associated with *V. pirina* resistance in the LG3 and LG7 of ‘Abbè Fétel’ (*P. communis*). Bouvier et al. [7] also identified a new *Rvp1* resistant to *V. pirina* in LG2 of ‘Navara’ (*P. communis*).

Since pear and apple belong to the same subfamily Pomoideae in Rosaceae, genome synteny has been frequently observed [8]. The genome sequencing of pear and apple revealed an almost equal number of genes in the two genomes [9,10]. Further, a comparison between pear and apple genomes has accelerated the identification of genes controlling traits of interest; particularly, the pear and apple scab (*V. inaequalis*) *R* genes located in the same homologous LG [4,7]. Terakami et al. [4] also suggested a comparison of the genetic location in LG could help understand genome synteny in scab resistance between pear and apple.

In a previous study, an interspecific hybrid pear, ‘Greensis’, was selected from seedlings derived from a cross between ‘Whangkeumbae’ (*P. pyrifolia*) and ‘Bartlett’ (*P. communis*) [11]. Because the paternal parent ‘Bartlett’ has resistance to *V. nashicola* [12], ‘Greensis’ displayed scab resistance when artificially inoculated with *V. nashicola* [11]. Therefore, we hypothesized that a mapping population derived from a cross with ‘Greensis’ would be ideal for identifying the *R* gene against *V. nashicola*. Herein, we performed an association mapping study to explore novel *R* genes, which confer resistance to *V. nashicola* using a pseudo-BC_1_ population derived from a cross between scab-resistant ‘Greensis’ [11] and scab-susceptible ‘Whasan’ [13].

## 2. Results

### 2.1. Evaluation of Pear Scab Resistance

Six weeks after the inoculation of *V. nashicola*, there were no disease symptoms on the leaves of the scab-resistant cultivar, ‘Greensis’. On the other hand, the inoculated leaves of the susceptible cultivar, ‘Whasan’, were wrinkled and chlorosis with sporulation was observed. Based on the Shay and Hough [14] and Chevalier et al. [15] studies, disease symptoms of the pseudo-BC_1_ individuals were divided into five classes. Thirty-nine and thirty-eight out of ninety-three pseudo-BC_1_ individuals showed resistance (hypersensitive response (HR) and chlorotic reaction) in 2016 and 2018, respectively. The remaining individuals showed sporulation and were regarded as susceptible individuals. The segregation ratios of scab-resistant to scab-susceptible individuals in 2016 and 2018 fitted the expected ratio (1:1) at a significance level of 5% (Table 1).

### 2.2. Construction of Genetic Linkage Maps

Figures S1 and S2 show genetic linkage maps of ‘Greensis’ and ‘Whasan’, respectively. These two genetic linkage maps consist of 17 LGs corresponding to the basic chromosome number of pear. The 17 LGs of ‘Greensis’ and ‘Whasan’ genetic maps were designated as G1 to G17 and W1 to W17, respectively. The genotyping-by-sequencing (GBS)-single nucleotide polymorphisms (SNPs) were grouped in the same homologous LG according to their chromosomal location. Each LG of ‘Greensis’ and ‘Whasan’ maps partially represent each pseudo-chromosome. The genetic map of ‘Greensis’ consisted of 673 GBS-SNPs and 52 simple sequence repeats (SSRs), which were distributed at a genetic distance of 1284.3 cM with an average marker density of 1.90 cM (Table S1). In the genetic map of ‘Whasan’, 630 GBS-SNPs and 27 SSRs were distributed at a genetic distance of 1612.4 cM with an average marker density of 2.59 cM (Table S2). The genetic maps of ‘Greensis’ and ‘Whasan’ covered 73.3 and 68.7% of the pseudo-chromosomes of pear, respectively.

### 2.3. Identification of a Novel Scab Resistance Gene

**Figure 1:**
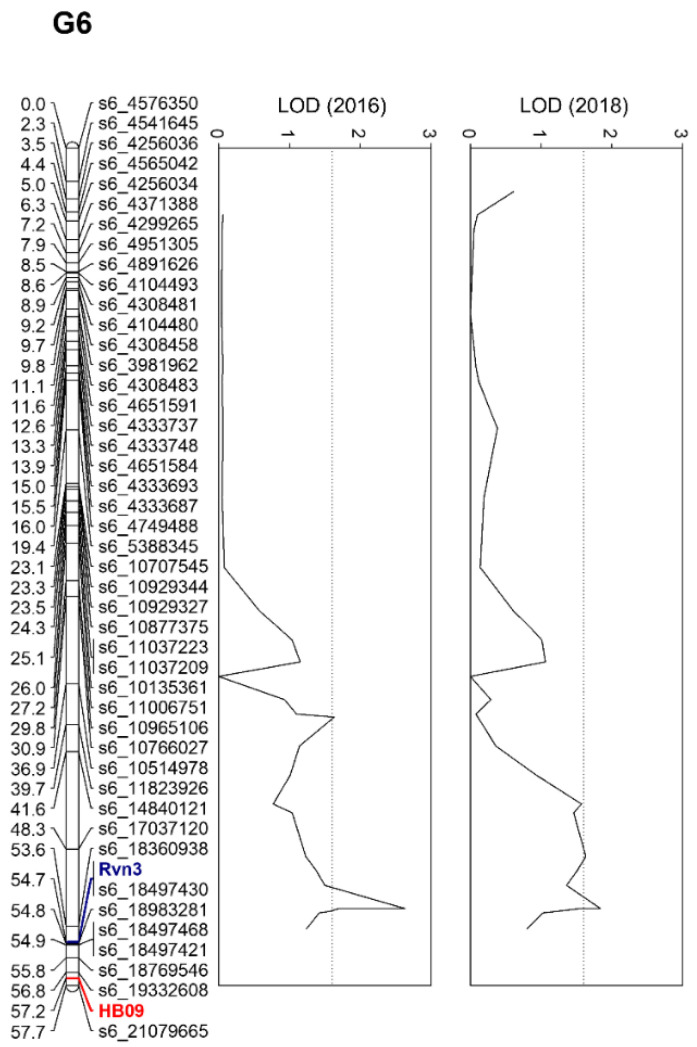
Linkage group (LG) 6 of ‘Greensis’ (G6) with the novel pear scab resistance gene, *Rvn3*, highlighted in blue. The number on the left of each marker indicates genetic distance (cM). The red marker is the SSR developed in bacterial artificial chromosome clone of *HcrVf2* regarded as candidate for apple scab resistance gene (*Vf*) [17]. The two graphs on the right represent the logarithm of odd (LOD) for each locus in 2016 and 2018.

Based on the Kruskal–Wallis test and interval mapping results, 14 loci were identified as significant markers associated with scab resistance. The logarithm of odd (LOD) thresholds in G6 were 1.6 and 1.5 in 2016 and 2018, respectively (*p* = 0.05). All 14 loci had a segregation type of <lm × ll> in ‘Greensis’ × ‘Whasan’ (Table S3). Twelve out of the fourteen loci were commonly associated with scab resistance in 2016 and 2018. These common genetic loci, including 11 SNPs and 1 SSR (HB09), were located at the distal end of G6 corresponding to a pseudo-chromosome 6. Higher LOD scores were confirmed between s6_17037120 and s6_18497564, and the SNP, s6_18497564, displayed higher phenotypic variance for the two years. For that reason, s6_18497564 (54.68 cM) was designated as *Rvn3* (Figure 1) with reference to the nomenclature for the apple scab resistance gene [16]. Interestingly, the HB09 was closely linked with *Rvn3*.

The LOD thresholds for the other 16 LGs ranged from 1.6 to 1.9 (Figures S3 and S4). There were loci in G3, G8, G13, G14, and G17 where the LOD scores were higher than the LOD threshold in each LG (Figure S3), but these loci were not significant in 2018 (Figure S4).

### 2.4. Insertion and Deletion Related with Scab Resistance

**Figure 2:**
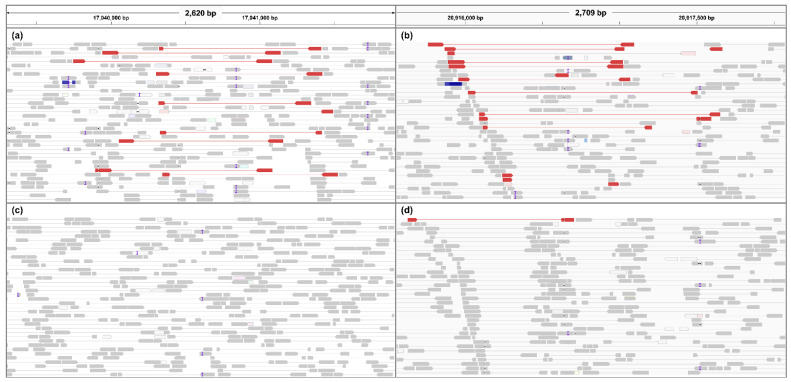
Alignment results of paired reads in ‘Greensis’ (**a**,**b**) and ‘Whasan’ (**c**,**d**). Gray reads are a match for the reference genome. Red reads indicate insertions that are larger than expected. Empty reads (colored white) indicate that none of reads are aligned to the reference genome. The ‘I’ colored purple is a small InDel variation.

Using the resequencing data of ‘Greensis’ and ‘Whasan’, we found insertion and deletion (InDel) structures based on the physical location of the loci significantly associated with scab resistance (Figure 2). The InDel structures of ~1770 and ~1370 bp in pseudo-chromosome 6 were inserted in ‘Greensis’, while they were deleted in ‘Whasan’. The InDel structures of ~1770 and ~1370 bp were found around the significant loci s6_17037120 and s6_21079665, respectively. In the homology analysis using the nucleotide sequences inserted in ‘Greensis’ (Supplementary file S1), four genes related to disease resistance in apples and pears were identified (Table 2). There was an apple scab resistance gene, namely *Malus floribunda* clone M18-6Bs Vf apple scab resistance protein HcrVf2-like gene. This gene showed more than 92% sequence similarity with the partial sequence of the inserted region in ‘Greensis’. The remaining two genes were likely serine/threonine protein kinase (XM_029106090.1) and rust resistance kinase Lr10-like (XM_008356659.3) of *M. domestica*. These two genes covered the InDel sequence of ~280 bp, respectively, and showed more than 84% similarity to the InDel sequences. In addition, there was a disease resistance protein RPM1-like gene with 97.91% sequence similarity in the InDel structure of ~1370 bp.

## 3. Discussion

*P. communis* possesses a single dominant homozygous gene controlling resistance to *V. nashicola* [3]. The pseudo-BC_1_ was derived from a cross between the scab-resistant cultivar ‘Greensis’ (*P. pyrifolia* cv. Whangkeumbae × *P. communis* cv. Bartlett) and the scab-susceptible cultivar ‘Whasan’ (*P. pyrifolia*). It was assumed that the maternal parent ‘Greensis’ has inherited *V. nashicola* resistance from its paternal progenitor ‘Bartlett’. Therefore, the ratio of resistant to susceptible pseudo-BC_1_ individuals was expected to be 1:1. Evaluating scab resistance in the pseudo-BC_1_ population indicated that the observed ratio of resistant to susceptible individuals fitted the expected ratio of 1:1 (*p* > 0.05) that was achieved in 2016 and 2018 (Table 1). These results suggest that resistance to *V. nashicola* in ‘Greensis’ is controlled by a single dominant gene.

To identify scab resistance loci, genetic linkage maps of maternal and paternal parents were constructed using GBS-SNPs and SSRs (Figures S1 and S2). The physical location of GBS-SNPs allowed us to designate LG as a pseudo-chromosome. Moreover, the anchored SSRs showed co-linearity with previously constructed genetic linkage maps of pear and apple [18,19,20]. Therefore, our genetic maps partially represent the physical location of the pear genome and they are suitable for comparative analysis between genera.

From the genetic linkage maps and phenotype data assessed for two years, we identified a new *Rvn3* conferring resistance to *V. nashicola* in the LG6 of ‘Greensis’ (G6) map (Figure 1 and Table S3). The genetic location of the *Rvn3* is different from that of the other pear scab *R* genes (i.e., *Vnk* in LG1 and *Rvn2* in LG2), and the pear scab *R* gene located in LG6 has not been identified until now. However, previously reported pear scab *R* genes displayed an orthologous relationship with apple scab *R* genes [4,7]. Furthermore, apple scab *R* genes located in LG1 and LG6 were considered paralogs [17]. Remarkably, HB09, which is closely linked to the apple scab *R* gene, *Rvi14* [21], is also closely linked to *Rvn3* with a genetic distance of 2.5 cM (Figure 1). Broggini et al. [17] developed the HB09 from a bacterial artificial chromosome (BAC) clone, which was isolated from hybridization with *HcrVf2*, a firstly cloned apple scab *R* gene [22], and Broggini et al. [17] confirmed that *HcrVf2* paralogs were distributed in LG1 and LG6. Therefore, LG6 in pears and apples may contain orthologous *R* genes to *Venturia* spp.

To demonstrate the orthologous relationship between the pear and apple scab *R* genes, the genomic structures of ‘Greensis’ and ‘Whasan’ on pseudo-chromosome 6 were compared. As a result, interesting insertions of ~1770 and ~1370 bp between mapped SNPs, s6_17037120 and s6_21079665, associated with *V. nashicola* resistance were detected in the downstream and upstream regions of *Rvn3*, respectively. The homology between the inserted sequence and *HcrVf2* paralog (Table 2) suggested that *Rvn3* may be an ortholog of *Rvi14*.

Interestingly, there were disease-resistance protein RPM1-like sequences in the insertion of ~1370 bp. The RPM1 protein is known to activate HR in response to pathogen invasion [23]. Moreover, resistant individuals of the pear pseudo-BC_1_ (‘Greensis’ × ‘Whasan’) showed two resistant phenotypes, including HR and chlorotic-type reactions. Thus, we thought that the RPM1-like gene could contribute to the control of the scab resistance in ‘Greensis’.

In addition, a gene with serine/threonine protein kinase activity was detected in the insertion region. Park et al. [24] and Jiang et al. [25] observed that *V. nashicola* penetrates the cuticle layer and develops hyphae in the intercellular region. The serine/threonine kinase activity of the intercellular protein kinase domain is one of the characteristics of receptor genes [26,27]. Therefore, it was assumed that certain receptor genes recognize the growth of *V. nashicola* hyphae in the intercellular region, and the protein kinases activate signal transduction in the cytoplasm to confer *V. nashicola* resistance.

Taken together, the novel pear scab resistance gene, *Rvn3*, was identified in LG6 of the interspecific hybrid pear ‘Greensis’. Based on the 1:1 segregation ratio of resistant to susceptible pseudo-BC_1_ individuals, a single dominant *Rvn3* was demonstrated to confer *V. nashicola* resistance. Since the ‘Greensis’ × ‘Whasan’ is a pseudo-BC_1_ population, based on our findings, the pyramiding strategy will facilitate the breeding of a pear cultivar with durable resistance to *V. nashicola*.

## 4. Materials and Methods

### 4.1. Plant Materials

A pseudo-BC_1_ population derived from a cross of ‘Greensis’ (*P. pyrifolia* × *P. communis*, a scab-resistant maternal plant) and ‘Whasan’ (*P. pyrifolia*, a scab-susceptible paternal plant) was used in the present study. Seedlings derived from ‘Greensis’ × ‘Whasan’ were grafted on *P. calleryana* and their scab resistance was screened.

### 4.2. Inoculation with V. nashicola and Scoring of Symptoms

Scab resistance was evaluated in the parents and 93 individuals of ‘Greensis’ × ‘Whasan’ in 2016 and 2018. Using *V. nashicola*-infected leaves collected in an orchard of the Pear Research Station, National Institute of Horticultural and Herbal Science in Korea (35°01′27.9″ N, 126°44′44.5″ E), a *V. nashicola* conidial suspension (5 × 10^5^ conidia·mL^−1^, 0.1% sucrose) was prepared. Droplets of the conidial suspension (2 μL) were placed on the upper epidermis of six fully expanded young leaves from two seedlings of each. The droplets were dried, and the inoculated leaves were wrapped in a plastic bag containing 1 mL of distilled water. The environmental conditions were maintained under the following conditions for 48 h: 20 °C, 70% relative humidity, and dark conditions. After that, 16 h of light periods were held with a light intensity of 108.10 μmol∙m^−2^∙s^−1^. Scab symptoms were evaluated at 6 weeks after inoculation. The symptoms were scored using 5 classes [14,15]: No visible symptoms (1), HR showing pin-point resistance reaction without sporulation (2), chlorotic wrinkled and/or necrotic reaction without sporulation (3), chlorotic reaction with sparse sporulation (4), and abundant sporulation lesion (5). Seedlings, which showed symptoms of classes 1 to 3, were regarded as scab-resistant. On the other hand, classes 4 and 5 were considered as scab-susceptible symptoms. For subsequent association analysis, the resistant and susceptible individuals were finally scored as 1 and 0, respectively.

### 4.3. Statistical Analysis

The chi-square test was performed to compare the segregation ratio of resistant to susceptible individuals in the pseudo-BC_1_ with an expected segregation ratio of 1:1 at a 5% significance level.

### 4.4. Linkage and QTL Analysis

Genetic maps of ‘Greensis’ and ‘Whasan’ were constructed using JoinMap 5, and a pseudo-testcross strategy was employed [28]. The genotype data consisting of 5120 GBS-SNPs and 89 SSRs for the parent cultivars and their 93 pseudo-BC_1_ progenies [29] were used. LGs were determined with a minimum LOD threshold of 7.0. The relative distances between markers were calculated by regression mapping and Kosambi’s mapping function.

Phenotype and genotype data of pseudo-BC_1_ individuals were loaded to MapQTL 6. The scab-resistance gene was decided by performing the Kruskal–Wallis test [30] and interval mapping with 1000 permutation tests. The LOD threshold was determined to identify significant loci at *p* = 0.05. MapChart 2.3 was applied to visualize genetic linkage maps and the novel scab resistance gene.

### 4.5. Resequencing and InDel Detection

Genomic DNA of ‘Greensis’ and ‘Whasan’ was isolated from young leaves using the DNeasy Plant Mini Kit (Qiagen, Hilden, Germany) according to the manufacturer’s instructions. DNA libraries with approximately 550 bp of short inserts were constructed using the Truseq DNA PCR-Free kit (Illumina, San Diego, CA, USA) and sequenced with paired-ends using the Illumina Hiseq 2500 platform. Base calling of 101 bp at each end was performed using CASAVA v1.8.2 and the reads were trimmed using SolexQA [31] and Cutadapt [32]. The genome assembly data of *P. bretschneideri* cv. Dangshansuli v1.1 [33] were used as a reference for the alignment of the trimmed reads. The Burrows-Wheeler Aligner was employed to align the reads [34] using the default option. BAM files were produced using SAMtools [35] and Picard (http://broadinstitute.github.io/picard, Accessed 16 October 2017) [36] to remove PCR duplicates. The BAM files were loaded into the Integrative Genomics Viewer (IGV, [37]) to detect sequence variations between ‘Greensis’ and ‘Whasan’.

Sequences of InDels, which existed in ‘Greensis’ and ‘Whasan’ genomes, were extracted in IGV, and homology with public genes was analyzed using the blastn function in the National Center for Biotechnology Information (NCBI).

## Figures and Tables

**Table 1:** Segregation of scab-resistant and scab-susceptible individuals in ‘Greensis’ × ‘Whasan’ after artificial inoculation of *Venturia nashicola* in 2016 and 2018.

Year	No. of Individuals		Expected Segregation Ratio	χ^2^-Value	*p*
	Resistant	Susceptible			
2016	39	54	1:1	2.42	0.12
2018	38	55	1:1	3.11	0.08

**Table 2:** Results of the sequence similarity analysis of the insertion region in pseudo-chromosome 6 of ‘Greensis’.

Gene Description	Accession No.	Query Cover (%)	E-Value	Similarity (%)
*Malus domestica* probable serine/threonine protein kinase IRE	XM_029106090.1	16	3.00 × 10^−72^	84.42
*Malus domestica* rust resistance kinase Lr10-like	XM_008356659.3	12	1.00 × 10^−70^	89.73
*Malus floribunda* clone M18-6Bs Vf apple scab resistance protein *HcrVf2*-like gene, complete sequence	EU794447.1	8	2.00 × 10^−53^	92.21
*Pyrus* × *bretschneideri* disease resistance protein RPM1-like	XM_018651218.1	40	6.00 × 10^−138^	97.91

## Supplementary Materials

The following are available online at https://www.mdpi.com/article/10.3390/plants10122632/s1, Figure S1: Genetic linkage map for ‘Greensis’ (*Pyrus pyrifolia* × *P. communis*). The linkage groups (LGs) of ‘Greensis’ are designated G1 to G17 and each LG number corresponds to a pseudo-chromosome number of the pear reference genome. The number on the left of each marker indicates genetic distance (cM). Markers highlighted with black are GBS-SNPs. SSRs from pear and apple are highlighted with green and red, respectively, Figure S2: Genetic linkage map for ‘Whasan’ (*Pyrus pyrifolia*). The linkage groups (LGs) of ‘Whasan’ are designated W1 to W17 and each LG number corresponds to a pseudo-chromosome number of the pear reference genome. The number on the left of each marker indicates genetic distance (cM). Markers highlighted with black are GBS-SNPs. SSRs from pear and apple are highlighted with green and red, respectively, Figure S3: Logarithm of odd distributions in the linkage groups of ‘Greensis’ map. Association analysis was performed using genotype data of ‘Greensis’ map and phenotypic data evaluated in 2016, Figure S4: Logarithm of odd distributions in the linkage groups of ‘Greensis’ map. Association analysis was performed using genotype data of ‘Greensis’ map and phenotypic data evaluated in 2018, Table S1: The number of markers, total genetic distance, and marker density of the linkage group (LGs) in ‘Greensis’ map, Table S2: The number of markers, total genetic distance, and marker density of the linkage groups (LGs) in ‘Whasan’ map, Table S3: Pear scab (*Venturia nashicola*) resistance loci in linkage group 6 of ‘Greensis’ detected by Kruskal–Wallis test and interval mapping in 2016 and 2018, Supplementary file S1: Two inserted sequences of ‘Greensis’ detected from resequencing data, Supplementary file S2: Sequence similarity between two InDels detected in ‘Greensis’ and disease resistance genes described in Table 2 using blastn in National Center for Biotechnology Information.
