# Supplementary material for: A Novel Pear Scab (Venturia nashicola) Resistance Gene, Rvn3, from Interspecific Hybrid Pear (Pyrus pyrifolia × P. communis)
Source: Plants (Basel). 2021 Nov 30;10(12):2632. doi: 10.3390/plants10122632 (PMC8705610; doi:10.3390/plants10122632)
Supplement: Supplementary file 1 [file plants-10-02632-s001.zip › Supplementary file 1.pdf]

Chr6:17039700-17041450

ACTTRGTCTCCTCTCCTGAGCTTCATTTCCATWTAAAGTACATCGAAAATGGTTGAGAATTGAGAAA
GTTATGAAGTTTTAAAAAATTTCCAGAAATCCGACGACTTTTTCCAAAATTCGGYGAACCAGACGGC
GGCARYGAGTGGTTGTCGTGCACARGTCCGTGACTTCGTGTAGAATGTTTTYGTATATTTAAACACT
TCGTTTGAGCAAACTATGGGRGGATTTTCCTAGCCTTTTAGTATATGTCCTAATTAATTAATTACTATAG
TTGTTTCAYATATARGAGAGAATTACCCCGAGRAGGTACGTGGACAAGCAAGGCTAGRAGGTTACGA
TTCGACTATGTATCAGTGAGTGGGCAWTTGTTTTTATATATTATATATAARGTTTTATARTTTCAACAAA
TGCTTTTGGATTGATTTTAGGGAAYTTTAACGAAAAGCTCMCAGTACTGTTTACTTTAAYGAAAAAC
CACATTTTTACACTAAAAAATCAATCATGGTACTATTCACTTTACCATTTATTTTGTCCTTATCGTTAAA
ACTCAAAGTTTTCAAGCTCTTTTCATTAGTTTTCCTTTGATTTTATGCCTATCATGCCATGTTTTATTTA
ATTTTAAAAATGCCATTRTATTGTTGAGATTTATATATTGTCATGGCATGTTCATATACATTATACCTACT
CATCATGCATGCTTGCACCAGTGTGTTAGGAAAAACTTAGAGTACTCAACTCTARCACAAGTGTTGG
AGAGACAATACAAGTAAACAAATTACTTGTATTACACAAACAAAATATTACAACACTCTCAAAGGAC
ACTTTTAGAAGCAATGACACTCACTTACTTTCTAGAGACACACTCTAGATCACTCACACTACTACAA
GACTATTCTTGCTTCTCACTCTYACTTGATTGCTTGCTTGCTTGATTACAAGCTTGCTTGGTTGGTTAC
ATAGCACCACACCCATATTTATAGGGCGTGTTTGCCGACTTTGGACATTGCTAGATCATTCTAGCTGC
TAATCATGCATGCCACCGGCATATGGCTAATAGTCATATAATATTACAACTTGCTAGAATTATCTAGCTT
ATTATGCATGCCCACCRACTTTAACATGCAAATTTGAACTAGAATGGTCTAGTTCATTTGCAGCTGGA
CAAAGTCTTCTAGAAGCTTCTACCTTCCAAGCTTGCTGCTTGTATTTGCTAGACTTTTCTAGAATCCA
AACACCAATTGGGCTGGTGTTGATCTTCACAGTGTAGAACTCGCCTNNNNNNNNNNCYAGGGCCAG
GCTTCACGTATATGCTCACATCGCACCACACGCTCACCTTGGATCCATTGTAGGTGCCAATCTTYATM
TGYGTCTGCATTCCTCCTAGGCTGCGCAACTGGTTTGGTCTGTACAAGATCAATAGAGTAGGCGGTA
AGGGTAGTTACACCTCACCACCATGAACGACAAAGAGATGGCGSTAATGATCGAATTGTTTGGCCTT
RGATAAAATGGGGACTTTACAGKGAAAAGCGGCTACCATAGAGCTCACCTCATTCGGGTCCCTAGTG
AGGAATTTCATCCTYATTCCTCTCACCAGATTGATACGAAGGTTTGGCACTTGATTTGGAATGCAAAA
GTGRTCCCRAAGATATCTAATTTTCTGTGGAGGGCAACATCTACTTATTTACCAACCAGGTCGACGCT
TGCTCATCGACACCTCACTACCAACCTTATTTCCTATTTGTAATTCACAA

Chr6:20915750-20917115

ACTTGGGAGGGATTCCTGTTGTGTGTTTAGTGAGTTATTTACATACAATCTGTGAAGCTATAGGT
AAGTGCTGTCTTATTTAATTTAAASAAAAAGGATCATKTTTTATGAGAAATATTAGTGTTGTTTT
GTTATATGTTTAATTCAAATCAACAAATATACCGTTGTTATGAAAGAAATCCGATTGTTTGTGAT
TGTTAGATTTATGGAGTAATTAGTCCATTTGGATAATGCCAGTCATTTCTCTATGCCGCATGAGG
GGATGTAGATCAAACTTTGTTTGAATTGAGTTTGGCTCCATATTAAAAACTGCTAAAGATTGAA
GATTGTCTTGATATCAAGTTTAGCAATGAATTATTAGATGAGCAAACTCRGCCCCAGTTTTTTTT
TTTTACATACACGTATCAATCGGACTGAGATTTTTTGTTTYACCTTGCCTGGATGTGTTGCAGGT
GCRGGGTTGTTCGAGAAAAAAAGGTTTTAAAGGATCCTTTGCAGATGCAGGATCTTCTGCTAAG
GTATGGGAATTCCTATTATTAAATGATAGCATTGCAATCCGAGTGTAATTAGTTTAGTTTTTTTGT
GAAATGCTATTTACAGTGTCAGAATGTTTCTTTGAATTCAGTTGTTAAGGTTTCTTTTTATATTTT
CCCCTTTTTAGACATTGCGGTTTTGTCCGGAGTGCACAGTTTGGTTGTCTGCATGTTGAAGAGG
CTGCGAGGAAAGGATGATGGTACGGGAACTTGAATGCAAAAATACTACTTTTACGTTATTCATT
CATTATATNGTGTTCACTMTTTCTTCCTCTGCAGTCAYTAATGYTGGAGTAGCTGGATGTTGCAC
TGGTCTTGMTCTTAGTTTTCCAGGTATCCATCGTATTAYAGAGTTCGATAATTTATAAATTARCAA
TAAACTCATTGACTGTGCTATTAAAATTTAAATTCTTAATATCTATAGAACTCCCGATTTTTGTTC
TCTCTCATTTTTGGATTGTTATAACGAGCAAGTCTTTTCCTTGCTAATCAATAAAACAGTTTCTG
TACAGTCTATTGTATCTTTAATTTAGAGTACCTTATTTAATTTGCAGGTGCACCTTAGGCWCTTCT
ACAAAGCTGTCTCACTTTTRGGGCATTCTCATTTGTTATTGAAGGCCTATACAAGCAGCAGCCG
GCYTTAGCACAACCATTTTCTGTGAGGAAGAAAAGTGAGCAAGTTTGTCCCTTGGTGCTCCCC
CTTCAACTTTCACTCCCGGGAGAACTAAAAGCGGGTTTCATCTTCTTTTGCAATTCTTTTAAGA

ATCRCAAGAAGGGCACTTCTCACCCAGCCTAAGAAAAGGTTGTTCCAGAAAGGTGGAAAGTG
CACACCAC
