# Supplementary material for: A Novel Pear Scab (Venturia nashicola) Resistance Gene, Rvn3, from Interspecific Hybrid Pear (Pyrus pyrifolia × P. communis)
Source: Plants (Basel). 2021 Nov 30;10(12):2632. doi: 10.3390/plants10122632 (PMC8705610; doi:10.3390/plants10122632)
Supplement: Supplementary file 1 [file plants-10-02632-s001.zip › supplementary file 2.pdf]

PREDICTED: Malus domestica probable serine/threonine protein kinase IRE (LOC103439649), transcript variant X2, mRNA

Sequence ID: XM_029106090.1 **Length:** 4208 **Number of Matches:** 1

Range 1: 3669 to 3975 GenBank Graphics ▼ Next Match ▲ Previous Match

Score	Expect	Identities	Gaps	Strand
287 bits(155)	3e-72	260/308(84%)	22/308(7%)	Plus/Plus

```
Query  660   TGTT-AGGAAAAACTTAGAGTACTCAACTCTARCACAAGTGTTGGAGAGACAATACAAGT  718
             |||| |||||||| ||||||||| |||||||| |||||||||| ||||||||| ||||||
Sbjct  3669  TGTTGAGGAAAAATTTAGAGTACACAACTCTAACACAAGTGTTTGAGAGACAAAACAAGT  3728

Query  719   AAACAAATTACTTGTATTACACAAACAAAATATTACAACACTCT------CA-AGGACA  771
             ||||||||||||||||||||||| |||||||||||||||||||       || || ||||
Sbjct  3729  AAACAAATTACTTGTATTACACACACAAAATATTACAACACTCAAAAGGACACAAAGACA  3788

Query  772   CT-TTTAGAAGCAATGACACTCACTTACTTTCTAGAGACACACTCTAGATCACTCACACT  830
             |  ||||||||||||| ||||||||||||||||||||||| |||||||||||||||||||
Sbjct  3789  CACTTTAGAAGCAATGGCACTCACTTACTTTCTAGAGACAAACTCTAGATCACTCACACT  3848

Query  831   ACT-ACAAGACTATTCTTGCTTCTCACTCTYACTTGATTGCTTGCTTGCTTG----ATTA  885
              || ||||||||| |||| ||||||||||| |||||||||||||||||||||    ||||
Sbjct  3849  CCTCACAAGACTACTCTTACTTCTCACTCTCACTTGATTGCTTGCTTGCTTGCTTGATTA  3908

Query  886   CAAGCTTGCTTG--G-TTG---G-TTACATAGCACCACACCCATATTTATAGGGCGTGTT  938
             |   ||| ||||  | |||   | || || | || ||||| | ||||||||||  |||||
Sbjct  3909  CTT-CTTTCTTGTTGGTTGATTGCTTTCACAACATCACACACCTATTTATAGGTGGTGTT  3967

Query  939   TGCCGACT  946
             ||||||||
Sbjct  3968  TGCCGACT  3975
```

PREDICTED: Malus domestica rust resistance kinase Lr10-like (LOC103418544), mRNA

Sequence ID: XM_008356659.3 **Length:** 3198 **Number of Matches:** 1

Range 1: 9 to 231 GenBank Graphics ▼ Next Match ▲ Previous Match

Score	Expect	Identities	Gaps	Strand
281 bits(152)	1e-70	201/224(90%)	10/224(4%)	Plus/Minus

```
Query  670   AACTTAGAGTACTCAACTCTARCACAAGTGTTGGAGAGACAATACAAGTAAACAAATTAC  729
             |||||||||||| |||||||| |||||||||||||||||||| ||||| |||||||||||
Sbjct  231   AACTTAGAGTACACAACTCTAACACAAGTGTTGGAGAGACAAAACAAGCAAACAAATTAC  172

Query  730   TTGTATTACACAAACAAAATATTACAACACTC-------TCAAAGGACAC--TTTTAGAA  780
             |||||||||||  |||||||||||||||||||       ||||| |||||   |||||||
Sbjct  171   TTGTATTACACTCACAAAATATTACAACACTCAAAACACTCAAA-GACACAACTTTAGAA  113

Query  781   GCAATGACACTCACTTACTTTCTAGAGACACACTCTAGATCACTCACACTACTACAAGAC  840
             ||||||||||||||| |||||||||||||| ||||||||||||||||||| |||||||||
Sbjct  112   GCAATGACACTCACTCACTTTCTAGAGACAAACTCTAGATCACTCACACTCCTACAAGAC  53

Query  841   TATTCTTGCTTCTCACTCTYACTTGATTGCTTGCTTGCTTGATT  884
             ||||||||||||||||||| ||||| ||||||||||||||| ||
Sbjct  52    TATTCTTGCTTCTCACTCTCACTTGGTTGCTTGCTTGCTTGCTT  9
```

Malus floribunda clone M18-6Bs Vf apple scab resistance protein HcrVf2-like gene, complete sequence

Sequence ID: EU794447.1 **Length:** 2819 **Number of Matches:** 1

Range 1: 1943 to 2096 GenBank Graphics ▼ Next Match ▲ Previous Match

Score	Expect	Identities	Gaps	Strand
224 bits(121)	2e-53	142/154(92%)	0/154(0%)	Plus/Plus

```
Query  377   AGGGAAYTTTAACGAAAAGCTCMCAGTACTGTTTACTTTAAYGAAAAACCACATTTTTAC  436
             ||| || |||||||||||||||   |||||||| ||||||| ||||||||||||||||||
Sbjct  1943  AGGAAACTTTAACGAAAAGCTCCGGGTACTGTTCACTTTAACGAAAAACCACATTTTTAC  2002

Query  437   ACTAAAAAATCAATCATGGTACTATTCACTTTACCATTTATTTTGTCCTTATCGTTAAAA  496
             |||||||| |||||||||||||||||||||||| | ||||||||||||||||||||||||
Sbjct  2003  ACTAAAAAGTCAATCATGGTACTATTCACTTTATCCTTTATTTTGTCCTTATCGTTAAAA  2062

Query  497   CTCAAAGTTTTCAAGCTCTTTTCATTAGTTTTCC  530
             ||||||||||||||||  ||||||||||||||||
Sbjct  2063  CTCAAAGTTTTCAAGCCTTTTTCATTAGTTTTCC  2096
```

PREDICTED: Pyrus x bretschneideri disease resistance protein RPM1-like (LOC103962513), transcript variant X1, mRNA

Sequence ID: XM_018651218.1 Length: 4363 Number of Matches: 5

See 2 more title(s) See all Identical Proteins(IPG)

Range 1: 780 to 1066 GenBank Graphics ▼ Next Match ▲ Previous Match

Score	Expect	Identities	Gaps	Strand
505 bits(273)	6e-138	281/287(98%)	0/287(0%)	Plus/Plus

```
Query  1080  TTTGCAGGTGCACCTTAGGCWCTTCTACAAAGCTGTCTCACTTTTRGGGCATTCTCATTT  1139
             ||| |||||||||| ||||| |||||||||||||||||||||||| ||||||||||||||
Sbjct  780   TTTCCAGGTGCACCCTAGGCTCTTCTACAAAGCTGTCTCACTTTTGGGGCATTCTCATTT  839

Query  1140  GTTATTGAAGGCCTATACAAGCAGCAGCCGGCYTTAGCACAACCATTTTCTGTGAGGAAG  1199
             |||||||||||||||||||||||||||||||| |||||||||||||||||||||||||||
Sbjct  840   GTTATTGAAGGCCTATACAAGCAGCAGCCGGCTTTAGCACAACCATTTTCTGTGAGGAAG  899

Query  1200  AAAAGTGAGCAAGTTTGTCCCTTGGTGCTCCCCCTTCAACTTTCACTCCCGGGAGAACTA  1259
             ||||||||||||||||||||||||||||||||||||||||||||||||||||||||||||
Sbjct  900   AAAAGTGAGCAAGTTTGTCCCTTGGTGCTCCCCCTTCAACTTTCACTCCCGGGAGAACTA  959

Query  1260  AAAGCGGGTTTCATCTTCTTTTGCAATTCTTTTAAGAATCRCAAGAAGGGCACTTCTCAC  1319
             |||||||||||||||||||||||||||||||||||||||| |||||||||||||||||||
Sbjct  960   AAAGCGGGTTTCATCTTCTTTTGCAATTCTTTTAAGAATCGCAAGAAGGGCACTTCTCAC  1019

Query  1320  CCAGCCTAAGAAAAGGTTGTTCCAGAAAGGTGGAAAGTGCACACCAC  1366
             |||||||||||||||||||||||||||||||||||||||||||||||
Sbjct  1020  CCAGCCTAAGAAAAGGTTGTTCCAGAAAGGTGGAAAGTGCACACCAC  1066
```

Range 2: 659 to 732 GenBank Graphics ▼ Next Match ▲ Previous Match First Match

Score	Expect	Identities	Gaps	Strand
137 bits(74)	3e-27	74/74(100%)	0/74(0%)	Plus/Plus

```
Query  659   AGACATTGCGGTTTTGTCCGGAGTGCACAGTTTGGTTGTCTGCATGTTGAAGAGGCTGCG  718
             ||||||||||||||||||||||||||||||||||||||||||||||||||||||||||||
Sbjct  659   AGACATTGCGGTTTTGTCCGGAGTGCACAGTTTGGTTGTCTGCATGTTGAAGAGGCTGCG  718

Query  719   AGGAAAGGATGATG  732
             ||||||||||||||
Sbjct  719   AGGAAAGGATGATG  732
```

Range 3: 533 to 597 GenBank Graphics ▼ Next Match ▲ Previous Match First Match

Score	Expect	Identities	Gaps	Strand
121 bits(65)	3e-22	65/65(100%)	0/65(0%)	Plus/Plus

```
Query  1     ACTTGGGAGGGATTCCTGTTGTGTGTTTAGTGAGTTATTTACATACAATCTGTGAAGCTA  60
             ||||||||||||||||||||||||||||||||||||||||||||||||||||||||||||
Sbjct  533   ACTTGGGAGGGATTCCTGTTGTGTGTTTAGTGAGTTATTTACATACAATCTGTGAAGCTA  592

Query  61    TAGGT  65
             |||||
Sbjct  593   TAGGT  597
```

Range 4: 594 to 660 GenBank Graphics ▼ Next Match ▲ Previous Match First Match

Score	Expect	Identities	Gaps	Strand
115 bits(62)	1e-20	65/67(97%)	0/67(0%)	Plus/Plus

```
Query  451   AGGTGCRGGGTTGTTCGAGAAAAAAGGTTTTAAAGGATCCTTTGCAGATGCAGGATCTTC  510
             |||||| |||||| ||||||||||||||||||||||||||||||||||||||||||||||
Sbjct  594   AGGTGCAGGGTTGGTCGAGAAAAAAGGTTTTAAAGGATCCTTTGCAGATGCAGGATCTTC  653

Query  511   TGCTAAG  517
             |||||||
Sbjct  654   TGCTAAG  660
```

Range 5: 732 to 788 GenBank Graphics ▼ Next Match ▲ Previous Match First Match

Score	Expect	Identities	Gaps	Strand
95.3 bits(51)	2e-14	54/57(95%)	0/57(0%)	Plus/Plus

```
Query  811   GTCAYTAATGYTGGAGTAGCTGGATGTTGCACTGGTCTTGMTCTTAGTTTTCCAGGT  867
             |||| ||||| ||||||||||||||||||||||||||||| ||||||||||||||||
Sbjct  732   GTCATTAATGCTGGAGTAGCTGGATGTTGCACTGGTCTTGCTCTTAGTTTTCCAGGT  788
```
