# Supplementary material for: A Novel Pear Scab (Venturia nashicola) Resistance Gene, Rvn3, from Interspecific Hybrid Pear (Pyrus pyrifolia × P. communis)
Source: Plants (Basel). 2021 Nov 30;10(12):2632. doi: 10.3390/plants10122632 (PMC8705610; doi:10.3390/plants10122632)
Supplement: Supplementary file 1 [file plants-10-02632-s001.zip › Table S1.pdf]

Table S1. The number of markers, total genetic distance, and marker density of linkage groups (LGs) in 'Greensis' map.

LG	No. of SNPs	No. of SSRs	Total genetic distance (cM)	Marker density (cM)	Chromosome coverage (%)[z]
1	37	3	93.4	2.34	65.2
2	24	5	41.9	1.44	35.7
3	48	3	88.6	1.74	83.8
4	31	0	64.4	2.08	76.2
5	76	4	76.1	0.95	84.5
6	46	1	57.7	1.23	73.4
7	44	0	55.1	1.25	66.2
8	19	3	78.5	3.57	79.1
9	32	1	89.9	2.73	95.4
10	31	1	68.7	2.15	74.4
11	51	4	85.3	1.55	79.8
12	49	2	85.1	1.67	94.5
13	44	5	74.3	1.52	81.0
14	42	6	96.0	2.00	67.0
15	33	5	36.2	0.95	32.2
16	32	7	106.3	2.72	87.0
17	34	2	86.9	2.41	78.3
Total	673	52	1,284.3		
Avg.				1.90	73.7

[z] Calculated by dividing physical length of LG by full length of pseudo-chromosome.
