# Supplementary material for: A Novel Pear Scab (Venturia nashicola) Resistance Gene, Rvn3, from Interspecific Hybrid Pear (Pyrus pyrifolia × P. communis)
Source: Plants (Basel). 2021 Nov 30;10(12):2632. doi: 10.3390/plants10122632 (PMC8705610; doi:10.3390/plants10122632)
Supplement: Supplementary file 1 [file plants-10-02632-s001.zip › Table S2.pdf]

Table S2. The number of markers, total genetic distance, and marker density of linkage groups (LGs) in 'Whasan' map.

LG	No. of SNPs	No. of SSRs	Total genetic distance (cM)	Marker density (cM)	Chromosome coverage (%)[z]
1	22	2	51.6	2.15	69.6
2	63	2	97.9	1.51	61.7
3	48	2	143.2	2.86	95.3
4	38	1	111.7	3.02	74.5
5	41	2	96.2	2.24	85.1
6	46	1	93.4	1.99	45.5
7	36	1	112.9	3.05	71.8
8	31	0	70.7	2.28	85.4
9	45	2	98.6	2.10	74.7
10	46	4	130.0	2.60	87.3
11	22	3	80.4	3.22	81.4
12	40	1	71.0	1.73	23.0
13	19	0	67.1	3.53	82.8
14	17	1	33.6	1.87	31.2
15	42	2	107.2	2.44	50.5
16	23	0	120.0	5.22	68.8
17	53	3	126.9	2.27	80.2
Total	630	27	1,612.4		
Avg.				2.59	68.7

[z] Calculated by dividing physical length of LG by full length of pseudo-chromosome.
