# Supplementary material for: A Novel Pear Scab (Venturia nashicola) Resistance Gene, Rvn3, from Interspecific Hybrid Pear (Pyrus pyrifolia × P. communis)
Source: Plants (Basel). 2021 Nov 30;10(12):2632. doi: 10.3390/plants10122632 (PMC8705610; doi:10.3390/plants10122632)
Supplement: Supplementary file 1 [file plants-10-02632-s001.zip › Table S3.pdf]

Table S3. Pear scab (*Venturia nashicola*) resistance loci in linkage group 6 of 'Greensis' detected by Kruskal-Wallis test and interval mapping in 2016 and 2018.

Locus	Map position (cM)	Segregation type	2016				2018			
			K*	Significance[1]	LOD[2]	%Expl[3]	K*	Significance	LOD	%Expl
s6_4256034	4.97	<lm × ll>					2.783	*	0.61	3.0
s6_10514978	36.91	<lm × ll>	4.198	**			2.980	*		
s6_14840121	41.61	<lm × ll>	6.319	**	1.63	7.8				
s6_17037120	48.27	<lm × ll>	3.117	*	1.04	5.0	6.206	**	1.46	7.0
s6_18360938	53.64	<lm × ll>	6.745	***			3.051	*		
s6_18497421	54.90	<lm × ll>	6.327	**	1.42	6.8	3.443	*	1.03	5.0
s6_18497430	54.69	<lm × ll>	7.210	***	1.70	8.1	4.530	**	1.55	7.4
s6_18497468	54.88	<lm × ll>	5.786	**	1.56	7.4	3.394	*	1.21	5.8
s6_18497564	54.68	<lm × ll>	5.917	**	2.63	12.2	5.588	**	1.84	8.7
s6_18769546	55.82	<lm × ll>	4.739	**	1.24	6.0	2.980	*	0.80	3.9
s6_18983281	54.83	<lm × ll>	4.682	**			5.770	**		
s6_19332608	56.78	<lm × ll>	4.404	**			4.951	**		
HB09	57.20	<lm × ll>	4.062	**			5.408	**		
s6_21079665	57.66	<lm × ll>	4.739	**			6.753	***		

[1] *$P < 0.05$; **$P < 0.01$; ***$P < 0.005$.

[2] Logarithm of odd.

[3] Explained variance.
